# What is replication?

**DOI:** 10.1371/journal.pbio.3000691

**Published:** 2020-03-27

**Authors:** Brian A. Nosek, Timothy M. Errington

**Affiliations:** 1 Center for Open Science, Charlottesville, Virginia, United States of America; 2 University of Virginia, Charlottesville, Virginia, United States of America

## Abstract

Credibility of scientific claims is established with evidence for their replicability using new data. According to common understanding, replication is repeating a study’s procedure and observing whether the prior finding recurs. This definition is intuitive, easy to apply, and incorrect. We propose that replication is a study for which any outcome would be considered diagnostic evidence about a claim from prior research. This definition reduces emphasis on operational characteristics of the study and increases emphasis on the interpretation of possible outcomes. The purpose of replication is to advance theory by confronting existing understanding with new evidence. Ironically, the value of replication may be strongest when existing understanding is weakest. Successful replication provides evidence of generalizability across the conditions that inevitably differ from the original study; Unsuccessful replication indicates that the reliability of the finding may be more constrained than recognized previously. Defining replication as a confrontation of current theoretical expectations clarifies its important, exciting, and generative role in scientific progress.

## Abstract 2

What is replication? This Perspective article proposes that the answer shifts the conception of replication from a boring, uncreative, housekeeping activity to an exciting, generative, vital contributor to research progress.

## Introduction

Credibility of scientific claims is established with evidence for their replicability using new data [1]. This is distinct from retesting a claim using the same analyses and same data (usually referred to as *reproducibility* or *computational reproducibility*) and using the same data with different analyses (usually referred to as *robustness*). Recent attempts to systematically replicate published claims indicate surprisingly low success rates. For example, across 6 recent replication efforts of 190 claims in the social and behavioral sciences, 90 (47%) replicated successfully according to each study’s primary success criterion [2]. Likewise, a large-sample review of 18 candidate gene or candidate gene-by-interaction hypotheses for depression found no support for any of them [3], a particularly stunning result considering that more than 1,000 articles have investigated their effects. Replication challenges have spawned initiatives to improve research rigor and transparency such as preregistration and open data, materials, and code [4–6]. Simultaneously, failures-to-replicate have spurred debate about the meaning of replication and its implications for research credibility. Replications are inevitably different from the original studies. How do we decide whether something is a replication? The answer shifts the conception of replication from a boring, uncreative, housekeeping activity to an exciting, generative, vital contributor to research progress.

## Replication reconsidered

According to common understanding, replication is repeating a study’s procedure and observing whether the prior finding recurs [7]. This definition of replication is intuitive, easy to apply, and incorrect.

The problem is this definition’s emphasis on repetition of the technical methods—the procedure, protocol, or manipulated and measured events. Why is that a problem? Imagine an original behavioral study was conducted in the United States in English. What if the replication is to be done in the Philippines with a Tagalog-speaking sample? To be a replication, must the materials be administered in English? With no revisions for the cultural context? If minor changes are allowed, then what counts as minor to still qualify as repeating the procedure? More broadly, it is not possible to recreate an earthquake, a supernova, the Pleistocene, or an election. If replication requires repeating the manipulated or measured events of the study, then it is not possible to conduct replications in observational research or research on past events.

The repetition of the study procedures is an appealing definition of replication because it often corresponds to what researchers do when conducting a replication—i.e., faithfully follow the original methods and procedures as closely as possible. But the reason for doing so is not because repeating procedures defines replication. Replications often repeat procedures because theories are too vague and methods too poorly understood to productively conduct replications and advance theoretical understanding otherwise [8].

Prior commentators have drawn distinctions between types of replication such as “direct” versus “conceptual” replication and argue in favor of valuing one over the other (e.g., [9, 10]). By contrast, we argue that distinctions between “direct” and “conceptual” are at least irrelevant and possibly counterproductive for understanding replication and its role in advancing knowledge. Procedural definitions of replication are masks for underdeveloped theoretical expectations, and “conceptual replications” as they are identified in practice often fail to meet the criteria we develop here and deem essential for a test to qualify as a replication.

## Replication redux

We propose an alternative definition for replication that is more inclusive of all research and more relevant for the role of replication in advancing knowledge. Replication is a study for which any outcome would be considered diagnostic evidence about a claim from prior research. This definition reduces emphasis on operational characteristics of the study and increases emphasis on the interpretation of possible outcomes.

To be a replication, 2 things must be true: outcomes consistent with a prior claim would increase confidence in the claim, and outcomes inconsistent with a prior claim would decrease confidence in the claim. The symmetry promotes replication as a mechanism for confronting prior claims with new evidence. Therefore, declaring that a study is a replication is a theoretical commitment. Replication provides the opportunity to test whether existing theories, hypotheses, or models are able to predict outcomes that have not yet been observed. Successful replications increase confidence in those models; unsuccessful replications decrease confidence and spur theoretical innovation to improve or discard the model. This does not imply that the magnitude of belief change is symmetrical for “successes” and “failures.” Prior and existing evidence inform the extent to which replication outcomes alter beliefs. However, as a theoretical commitment, replication does imply precommitment to taking all outcomes seriously.

Because replication is defined based on theoretical expectations, not everyone will agree that one study is a replication of another. Moreover, it is not always possible to make precommitments to the diagnosticity of a study as a replication, often for the simple reason that study outcomes are already known. Deciding whether studies are replications after observing the outcomes can leverage post hoc reasoning biases to dismiss “failures” as nonreplications and “successes” as diagnostic tests of the claims, or the reverse if the observer wishes to discredit the claims. This can unproductively retard research progress by dismissing replication counterevidence. Simultaneously, replications can fail to meet their intended diagnostic aims because of error or malfunction in the procedure that is only identifiable after the fact. When there is uncertainty about the status of claims and the quality of methods, there is no easy solution to distinguishing between motivated and principled reasoning about evidence. Science’s most effective solution is to replicate, again.

At its best, science minimizes the impact of ideological commitments and reasoning biases by being an open, social enterprise. To achieve that, researchers should be rewarded for articulating their theories clearly and a priori so that they can be productively confronted with evidence [4,6]. Better theories are those that make it clear how they can be supported and challenged by replication. Repeated replication is often necessary to resolve confidence in a claim, and, invariably, researchers will have plenty to argue about even when replication and precommitment are normative practices.

## Replication resolved

The purpose of replication is to advance theory by confronting existing understanding with new evidence. Ironically, the value of replication may be strongest when existing understanding is weakest. Theory advances in fits and starts with conceptual leaps, unexpected observations, and a patchwork of evidence. That is okay; it is fuzzy at the frontiers of knowledge. The dialogue between theory and evidence facilitates identification of contours, constraints, and expectations about the phenomena under study. Replicable evidence provides anchors for that iterative process. If evidence is replicable, then theory must eventually account for it, even if only to dismiss it as irrelevant because of invalidity of the methods. For example, the claims that there are more obese people in wealthier countries compared with poorer countries on average and that people in wealthier countries live longer than people in poorer countries on average could both be highly replicable. All theoretical perspectives about the relations between wealth, obesity, and longevity would have to account for those replicable claims.

There is no such thing as exact replication. We cannot reproduce an earthquake, era, or election, but replication is not about repeating historical events. Replication is about identifying the conditions sufficient for assessing prior claims. Replication can occur in observational research when the conditions presumed essential for observing the evidence recur, such as when a new seismic event has the characteristics deemed necessary and sufficient to observe an outcome predicted by a prior theory or when a new method for reassessing a fossil offers an independent test of existing claims about that fossil. Even in experimental research, original and replication studies inevitably differ in some aspects of the sample—or units—from which data are collected, the treatments that are administered, the outcomes that are measured, and the settings in which the studies are conducted [11].

Individual studies do not provide comprehensive or definitive evidence about all conditions for observing evidence about claims. The gaps are filled with theory. A single study examines only a subset of units, treatments, outcomes, and settings. The study was conducted in a particular climate, at particular times of day, at a particular point in history, with a particular measurement method, using particular assessments, with a particular sample. Rarely do researchers limit their inference to precisely those conditions. If they did, scientific claims would be historical claims because those precise conditions will never recur. If a claim is thought to reveal a regularity about the world, then it is inevitably generalizing to situations that have not yet been observed. The fundamental question is: of the innumerable variations in units, treatments, outcomes, and settings, which ones matter? Time-of-day for data collection may be expected to be irrelevant for a claim about personality and parenting or critical for a claim about circadian rhythms and inhibition.

When theories are too immature to make clear predictions, repetition of original procedures becomes very useful. Using the same procedures is an interim solution for not having clear theoretical specification of what is needed to produce evidence about a claim. And, using the same procedures reduces uncertainty about what qualifies as evidence “consistent with” earlier claims. Replication is not about the procedures per se, but using similar procedures reduces uncertainty in the universe of possible units, treatments, outcomes, and settings that could be important for the claim.

**Fig 1:**
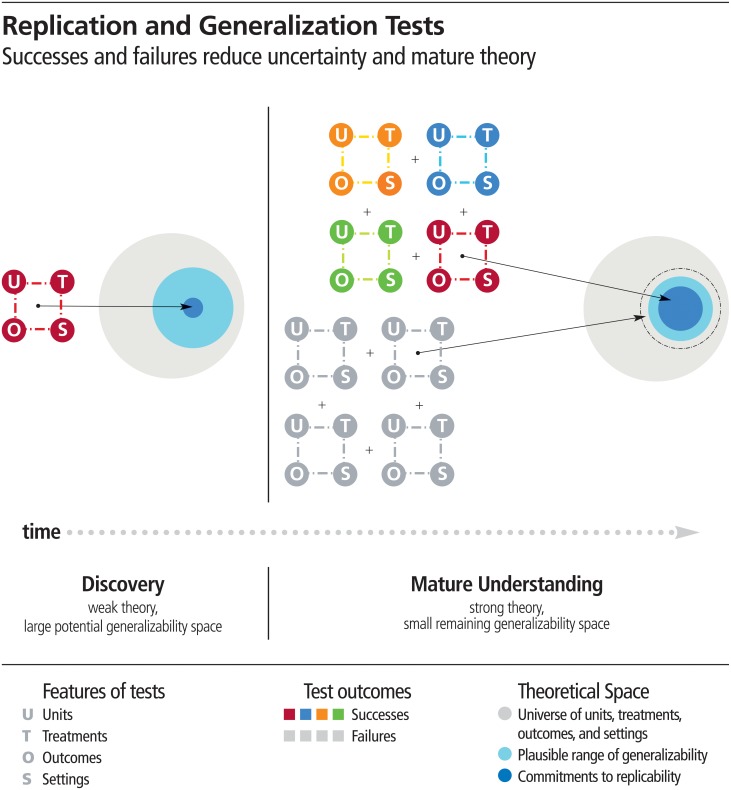
There is a universe of distinct units, treatments, outcomes, and settings and only a subset of those qualify as replications—a study for which any outcome would be considered diagnostic evidence about a prior claim. For underspecified theories, there is a larger space for which the claim may or may not be supported—the theory does not provide clear expectations. These are generalizability tests. Testing replicability is a subset of testing generalizability. As theory specification improves (moving from left panel to right panel), usually interactively with repeated testing, the generalizability and replicability space converge. Failures-to-replicate or generalize shrink the space (dotted circle shows original plausible space). Successful replications and generalizations expand the replicability space—i.e., broadening and strengthening commitments to replicability across units, treatments, outcomes, and settings.

Because there is no exact replication, every replication test assesses generalizability to the new study’s unique conditions. However, every generalizability test is not a replication. Fig 1‘s left panel illustrates a discovery and conditions around it to which it is potentially generalizable. The generalizability space is large because of theoretical immaturity; there are many conditions in which the claim might be supported, but failures would not discredit the original claim. Fig 1‘s right panel illustrates a maturing understanding of the claim. The generalizability space has shrunk because some tests identified boundary conditions (gray tests), and the replicability space has increased because successful replications and generalizations (colored tests) have improved theoretical specification for when replicability is expected.

Successful replication provides evidence of generalizability across the conditions that inevitably differ from the original study; unsuccessful replication indicates that the reliability of the finding may be more constrained than recognized previously. Repeatedly testing replicability and generalizability across units, treatments, outcomes, and settings facilitates improvement in theoretical specificity and future prediction.

**Fig 2:**
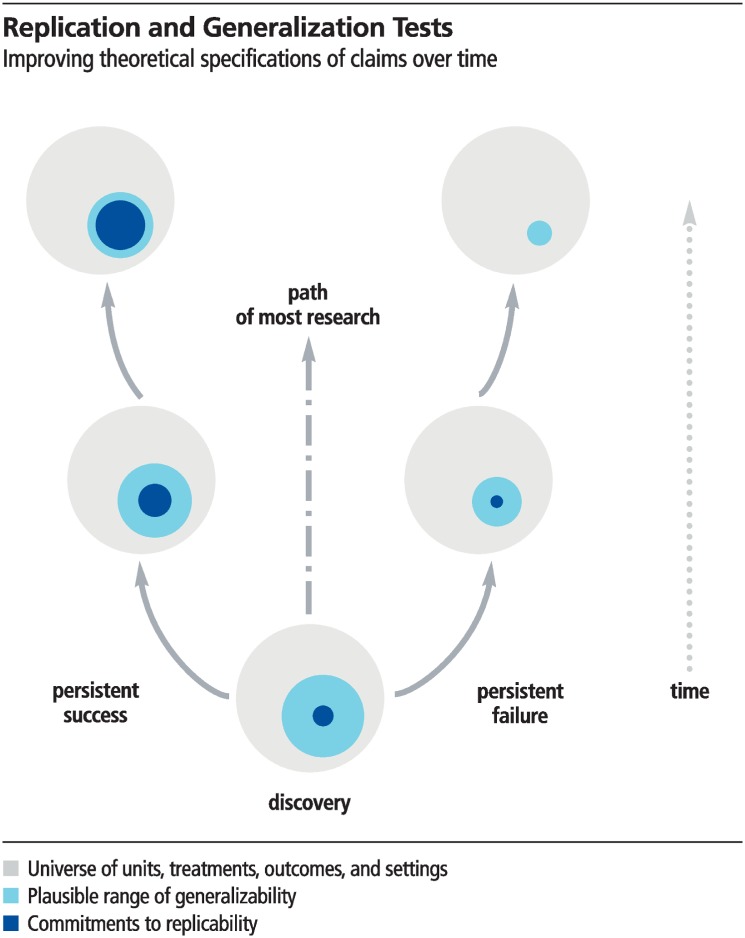
A discovery provides initial evidence that has a plausible range of generalizability (light blue) and little theoretical specificity for testing replicability (dark blue). With progressive success (left path) theoretical expectations mature, clarifying when replicability is expected. Also, boundary conditions become clearer, reducing the potential generalizability space. A complete theoretical account eliminates generalizability space because the theoretical expectations are so clear and precise that all tests are replication tests. With repeated failures (right path) the generalizability and replicability space both shrink, eventually to a theory so weak that it makes no commitments to replicability.

Theoretical maturation is illustrated in Fig 2. A progressive research program (the left path) succeeds in replicating findings across conditions presumed to be irrelevant and also matures the theoretical account to more clearly distinguish conditions for which the phenomenon is expected to be observed or not observed. This is illustrated by a shrinking generalizability space in which the theory does not make clear predictions. A degenerative research program (the right path) persistently fails to replicate the findings and progressively narrows the universe of conditions to which the claim could apply. This is illustrated by shrinking generalizability and replicability space because the theory must be constrained to ever narrowing conditions [12].

This exposes an inevitable ambiguity in failures-to-replicate. Was the original evidence a false positive or the replication a false negative, or does the replication identify a boundary condition of the claim? We can never know for certain that earlier evidence was a false positive. It is always possible that it was “real,” and we cannot identify or recreate the conditions necessary to replicate successfully. But that does not mean that all claims are true, and science cannot be self-correcting. Accumulating failures-to-replicate could result in a much narrower but more precise set of circumstances in which evidence for the claim is replicable, or it may result in failure to ever establish conditions for replicability and relegate the claim to irrelevance.

The ambiguity between disconfirming an original claim or identifying a boundary condition also means that understanding whether or not a study is a replication can change due to accumulation of knowledge. For example, the famous experiment by Otto Loewi (1936 Nobel Prize in Physiology or Medicine) showed that the inhibitory factor “vagusstoff,” subsequently determined to be acetylcholine, was released from the vagus nerve of frogs, suggesting that neurotransmission was a chemical process. Much later, after his and others’ failures-to-replicate his original claim, a crucial theoretical insight identified that the time of year at which Loewi performed his experiment was critical to its success [13]. The original study was performed with so-called winter frogs. The replication attempts performed with summer frogs failed because of seasonal sensitivity of the frog heart to the unrecognized acetylcholine, making the effects of vagal stimulation far more difficult to demonstrate. With subsequent tests providing supporting evidence, the understanding of the claim improved. What had been perceived as replications were not anymore because new evidence demonstrated that they were not studying the same thing. The theoretical understanding evolved, and subsequent replications supported the revised claims. That is not a problem, that is progress.

## Replication is rare

The term “conceptual replication” has been applied to studies that use different methods to test the same question as a prior study. This is a useful research activity for advancing understanding, but many studies with this label are not replications by our definition. Recall that “to be a replication, 2 things must be true: outcomes consistent with a prior claim would increase confidence in the claim, and outcomes inconsistent with a prior claim would decrease confidence in the claim." Many "conceptual replications" meet the first criterion and fail the second. That is, they are not designed such that a failure to replicate would revise confidence in the original claim. Instead, “conceptual replications” are often generalizability tests. Failures are interpreted, at most, as identifying boundary conditions. A self-assessment of whether one is testing replicability or generalizability is answering—would an outcome inconsistent with prior findings cause me to lose confidence in the theoretical claims? If no, then it is a generalizability test.

Designing a replication with a different methodology requires understanding of the theory and methods so that any outcome is considered diagnostic evidence about the prior claim. In practice, this means that replication is often limited to relatively close adherence to original methods for topics in which theory and methodology is immature—a circumstance commonly called “direct” or “close” replication—because the similarity of methods serves as a stand-in for theoretical and measurement precision. In fact, conducting a replication of a prior claim with a different methodology can be considered a milestone for theoretical and methodological maturity.

## Conclusion

Replication is characterized as the boring, rote, clean-up work of science. This misperception makes funders reluctant to fund it, journals reluctant to publish it, and institutions reluctant to reward it. The disincentives for replication are a likely contributor to existing challenges of credibility and replicability of published claims [14].

Defining replication as a confrontation of current theoretical expectations clarifies its important, exciting, and generative role in scientific progress. Single studies, whether they pursue novel ends or confront existing expectations, never definitively confirm or disconfirm theories. Theories make predictions; replications test those predictions. Outcomes from replications are fodder for refining, altering, or extending theory to generate new predictions. Replication is a central part of the iterative maturing cycle of description, prediction, and explanation. A shift in attitude that includes replication in funding, publication, and career opportunities will accelerate research progress.
